# Understanding parental preference for childcare: a cross-sectional study in Chongqing, China

**DOI:** 10.3389/fsoc.2024.1380334

**Published:** 2024-09-16

**Authors:** Boya Liu, Lunxin Liu, Hong Xu

**Affiliations:** School of Public Health, Chongqing Medical University, Chongqing, China

**Keywords:** infant care, preference for childcare, care models, Chinese family, the decision tree model

## Abstract

This study analyzed the factors influencing childcare preference and the relationship between childcare preference and childcare service demand scale, using data collected from a questionnaire survey of 3,921 parents of infants and toddlers in Chongqing, China. The results indicate that parents with higher incomes, higher education levels, older ages, multiple infants, and dual-career living in urban areas have a stronger preference for childcare. In the shared or grandparent care model, the childcare preference is not obvious. Parents of infants tend to choose childcare institutions that provide reception services, early education, and convenience services. Higher quality environmental facilities tend to reduce the preference of parents for childcare.

## 1 Introduction

Globally, fertility rates have been steadily declining for many years. In China, the fertility situation is changing in a similar way. Since the implementation of the family planning policy in the 1970s of the last century, China’s fertility rate has declined rapidly. Despite the government’s growing liberalization of the fertility policy in recent years, China’s fertility rate has remained low. A woman’s ability to work is one of several factors that can impact her fertility. Due to the impacts of urbanization and industrialization, modern professional women in China face multiple pressures, including childcare and employment, encompassing aspects such as time, childcare, and educational costs (Kang et al., 2022). With fathers generally less involved in the day-to-day care of infants, mothers, as the primary caregivers, need to juggle earning a living and caring for their family. Excessive domestic commitments can impede their performance in the labor market, affecting their income (Wu and Wang, 2017; Yu and Xie, 2018). Consequently, not only is the traditional mother-centric model of infant and toddler care becoming unsustainable, but it also contributes to a lower fertility desire among some women (Chen et al., 2023).

There are two main levels of preschool education in China: the first is the childcare provided by childcare institutions for infants and toddlers under the age of 3, and the other is the education and care services provided by kindergartens for children aged 3–6 years. Childcare services for infants and toddlers under the age of 3 in China have long been marginalized (Chen et al., 2023), but childcare affects fertility. Surveys indicate that up to 86.5% of families cite “lack of childcare” as the biggest obstacle to having a second child (CPPCC Network, 2016). Because of the lack of childcare services, most families resort to grandparents for help, making grandparental care a common practice in China (Chen et al., 2011; Du et al., 2019; Hong et al., 2021). According to a 2014 survey by the Shanghai Scientific Parenting Base, 73.4% of grandparents are the primary caregivers of their grandchildren, but 48.1% of these grandparents are in this role reluctantly; another survey shows that while 53% of children under three are cared for by grandparents, 70% of families are dissatisfied with this arrangement (Nyland et al., 2009; CPPCC Network, 2016). This indicates that parents of infants and toddlers are not satisfied with having grandparents take care of their infants and toddlers, but because the supply of childcare services in China is scarce and parents are too busy with work to take care of infants and toddlers, they have to maintain the status quo of grandparents taking care of infants and toddlers.

Childcare is the care and supervision of children while their parents are at work or busy with other activities, ensuring their safety and well-being (Conley, 2010). Childcare services play a positive role in the early development of infants, helping to unlock their potential and potentially influencing fertility rates (Campbell et al., 2014; Black et al., 2017). On the one hand, these services address the issues of unattended children in families and lessen the strain of mother-infant care on Chinese women. On the other hand, the government is keen to promote this professional model of care to encourage childbirth, ensuring that children are well cared for. International experience shows that childcare services, as a social welfare measure, can significantly boost female fertility rates, demonstrating a positive correlation between childcare services and fertility rates (Rindfuss et al., 2010; Karita and Kitada, 2018; Wood and Neels, 2019). To tackle the challenges in raising and educating infants and to balance the conflict between work and family, China has begun to prioritize the development of its long-marginalized childcare services. Soon after, a large number of nurseries offering childcare services started to pop up all throughout China. These nurseries may be run by the government or the private sector, but both had to charge a fee, and in most cases, the cost of childcare given by the government was less than that of childcare provided by the private sector. China’s childcare market is currently dominated by private daycare providers. In order to strengthen the development of the childcare service system, the Chinese government has released a number of policy documents since 2019. These documents clearly outline the objectives and tasks that must be accomplished. During the early stages of the system’s development, the government increased financial investment in childcare services, strengthened team-building and training programs, and strengthened supervision and evaluation procedures. Chongqing, one of China’s four municipalities directly under the central government, has a permanent population of 32,133,400, a birth population of 192,000, and a birthrate of 5.58. These figures indicate that Chongqing is among the provinces and cities with the lowest birthrate. The Chinese government’s ongoing focus on childcare services has led to improvements in Chongqing’s local childcare system as well. For example, the local government established uniform health evaluation standards for childcare facilities, formulated contracted service packages uniformly for the provision of health services to infants and young children, and purchased insurance for qualified childcare facilities, among other measures. As of right now, Chongqing has 541 registered qualified childcare facilities, with 98,900 person maximum capacity.

Some studies have found the influence of family background on childcare preferences, with higher family income levels or parents being in education longer, the more likely they are to choose childcare services (Huston et al., 2002; Dowsett et al., 2008). Some studies have found a relationship between job type and childcare preferences, with dual-income families being more likely to opt for childcare, especially when mothers are working full-time (Ertas and Shields, 2012; Hong et al., 2021); Studies have found that intergenerational care and community support can also reduce the preference of parents of infants and toddlers for childcare to a certain extent (Burchinal et al., 2008; Kim and Fram, 2009). Current research on childcare services in China is still in its developmental stage. The main areas of research include summarizing advanced practices and experiences from abroad (Liu, 2023; Suo and Wang, 2023), reviewing the development history and policy changes of childcare services in China (Yang, 2022; Liu and Liu, 2023), discussing the current state and issues of childcare service demand through survey data (Gao et al., 2020; Kang et al., 2023), and exploring the development and system construction of the childcare service industry (Pang et al., 2019; Chen et al., 2022). Most studies on childcare demand are based on a holistic consideration of surveyed families, usually classifying them into categories of “enrolled” and “not enrolled,” without considering the precondition of preference to enroll. As a result of this oversight, the conclusions of the study do not objectively reflect the actual childcare needs of families with childcare preferences. Therefore, the present study aimed: (1) to understand the preference of parents of infants and toddlers for childcare services in Chongqing, China; (2) to analyze the factors that affect the preference of childcare services.

## 2 Materials and methods

### 2.1 Study design and study population

This survey employed a multi-stage stratified random sampling combined with convenience sampling to select participants. From August 7th to 30th, 2022, a cross-sectional questionnaire survey was conducted in Chongqing, China. Chongqing was initially divided into four major sectors, each of which three districts or counties were randomly selected. Then, using convenience sampling, three community health service centers or township health clinics were chosen from each district or county. Based on the proportion of infants aged 0–3 years as announced by the government of Chongqing, the number of children under one year, 1–2 years, and 2–3 years was calculated. According to these proportions, infants from these three age groups were randomly sampled from the selected health centers or clinics. Subsequently, the parents of these children were contacted.

Chongqing, one of the four direct-controlled municipalities in China, is located in the southwest of the country, with a population of about 32 million. The inclusion criteria for participants were defined as follows: (1) aged between 18 and 59 years; (2) having at least one child under the age of three; (3) being able to understand and complete the questionnaire independently; and (4) proficiency in the use of online services such as computers, mobile phones, tablets, etc., as this survey used online questionnaires. In collaboration with the Population and Family Department of Chongqing Municipal Health Commission, eligible participants were contacted, and the survey was distributed and collected using the professional online survey platform “Wenjuanxing”.^1^ The study was conducted in accordance with the Declaration of Helsinki and approved by the Institutional Review Board (or Ethics Committee) of Chongqing Medical University for studies involving humans.

### 2.2 Data collection

We conducted an online questionnaire survey targeting fathers and mothers with at least one child under the age of three in 40 districts and counties of Chongqing. The survey primarily encompassed basic demographic information, preference for childcare, and a scale of demand for childcare services (SDCS).

The basic demographic information was divided into three categories: characteristics of families, characteristics of parents, and characteristics of infants. Characteristics of families included the following: residence (urban, rural), income (<4,000 Chinese Yuan, CNY, 4000–8,000 CNY, > 8,000 CNY), care model (parental care, grandparent care, shared care, other care). Characteristics of parents included parent’s age (18–29, 30–39, ≥40), education (primary/vocational, secondary, tertiary), employment status (both, just father, just mother, neither), reproductive will (willing, unwilling, not sure). Characteristics of infants included the following: infants’ number in the household (one, two, more than two), infants’ age (0–1, 1–2, 2–3), infants’ gender (male, female).

Preference for childcare was assessed by asking the question, “Depending on your current situation, whether you would like to send an infant under the age of 3 to a childcare facility?.” The answer choices were “yes” or “no.” The respondents who answered “yes” were expected to have a preference for childcare services.

The Scale of Demand for Childcare Services (SDCS) was developed by the Infant Socialization Education Research Group of the Hangzhou Preschool Teacher Education College at Zhejiang Normal University (Zhang, 2020). The scale was informed by materials such as the “Infant/Toddler Environment Rating Scale – Revised (ITERS-R)” and the “Shanghai Community Public Childcare Service Demand Survey Questionnaire” released by the Shanghai Preschool Education Information Department. Good validity, reliability, and suitability in China have been demonstrated for the SDCS (Hong et al., 2021; Sha et al., 2023). The initial version of this scale consists of 38 items in 6 dimensions: (a) environmental facilities, (b) nursing care, (c) early education, (d) teacher professionalism, (e) convenience services, and (f) home instruction. On the basis of the original 6 dimensions, the scale used in this survey adds 3 new dimensions according to local conditions and policy regulations: (a) reception services, (b) safety management, and (c) home instruction approach, and finally forms a scale with 9 dimensions and 49 items. Specific items under each dimension are shown in Table 1. The Likert scale is used, with 1 point representing “not needed at all” and 5 points representing “very much needed.” Higher scores indicate a greater demand for childcare services.

**Table 1:** The Scale of Demand for Childcare Services (SDCS).

Dimensions	Items
Environmental facilities	Environmental safety and hygiene.
	Adequate space allows for unrestricted activities.
	A variety of toys and books in sufficient quantities.
	Different activity areas for various stages of development.
	Surrounding environment has low noise levels and no pollution.
Reception services	Fees are commensurate with the childcare services provided.
	Establish good connections with families.
	Provide flexible childcare services.
	Organize a variety of parent–child activities.
	The number of infants in each class is reasonable.
	The ratio of teachers to students is reasonable.
Nursing care	The quality of meals is reliable, safe, and hygienic.
	When infants have allergies, taboos, or preferences for certain foods, alternative options could be provided.
	Cultivate the self-serving abilities of infants.
	Provide activities and supervision for infants who do not nap in the afternoon.
	Reasonably arrange sleep times.
	Ensure at least 2 h of outdoor activities every day.
Early education	Able to select appropriate educational content based on the age characteristics.
	Promote physical development of infants in activities.
	Promote cognitive ability development of infants.
	Assist infants in peer interactions.
Safety management	Equipped with medical facilities.
	Install video surveillance systems at the main entrances, and in the living and activity areas.
	Implement strict drop-off and pick-up policies.
	Be prepared for emergencies such as natural disasters, infectious disease isolation, and sudden violent incidents.
	Conduct regular safety drills.
Teacher professionalism	Caring, patient, attentive, and responsible.
	Friendly and liked by infants.
	Hold professional qualifications and certifications.
	Possess professional knowledge, abilities, and skills.
	Able to perceive children’s physical needs and emotional changes and respond accordingly. Communicate with parents properly.
	Continuously improve the level of education.
Convenience services	The location is close to the family’s residence.
	The location is close to the workplace.
	Temporary childcare services are available.
	Services are available during winter and summer vacations.
Home instruction content	Guide parents in learning about care.
	Guide parents in learning about cognitive development.
	Guide parents in learning about social development.
	Guide parents in learning about emotional development.
	Guide parents in learning about language development.
	Guide parents in learning about motor development.
Home instruction approach	Face-to-face consultations.
	Expert lectures.
	Online platforms.
	Telephone consultations.
	Parenting books.
	Home visits.

The original scale had a Cronbach’s Alpha coefficient of 0.918, while the modified scale used in this survey has a coefficient of 0.952, indicating good internal consistency. The Cronbach’s alpha values for each dimension are as follows: (a) 0.979, (b) 0.970, (c) 0.976, (d) 0.976, (e) 0.982, (f) 0.990, (g) 0.917, (h) 0.989, and (i) 0.955, with the original scale values being 0.865, 0.788, 0.813, 0.802, 0.721, and 0.879, due to the difference in sample size and the influence of the revised scale, the alpha values obtained this time are different from the alpha values of the original scale. However, the alpha values obtained this time can indicate an improvement in the reliability of the scale used in this survey.

### 2.3 Statistical analysis

All data were inputted into EpiData 3.1 and analyzed using STATA version 17.0, which included descriptive statistics, Chi-square test, logistic regression analysis, t-test, and decision tree analysis. According to a previous study, compared to using only a decision tree or linear classification (not both simultaneously), combining a decision tree with linear classification (such as logistic regression) in binary classification may yield better performance (Zhang et al., 2022).

The binary logistic regression analysis was employed to identify factors influencing parents’ preference for childcare. Variables that showed statistical significance in bivariate analysis and were identified as risk factors in existing literature were included in the model.

The IBM SPSS Modeler software was used to create the decision tree model, with the accuracy of the model identified through 10-fold cross-validation to avoid underfitting or overfitting problems (Blockeel and Struyf, 2001; Zhang et al., 2022). Decision tree analysis utilized the Chi-square Automatic Interaction Detection (CHAID) model to assess the factors impacting parents’ preference for childcare. Demographic characteristics, including characteristics of families, characteristics of parents, and characteristics of infants, were included in the model as independent variables. A *p*-value of ≤0.05 was considered statistically significant.

## 3 Results

### 3.1 Sociodemographics and preference for childcare

In this survey, 4,200 questionnaires were distributed, and after excluding invalid responses, 3,921 valid questionnaires were retrieved, resulting in a response rate of 93.36%. The survey covered the entire city of Chongqing, China. Over half of the surveyed families had a preference for childcare (52.7%). Most of these participants resided in urban areas (59.2%), with family average monthly incomes falling below 4,000 CNY (36.3%) and between 4,000 and 8,000 CNY (44.1%). The most common childcare model among the surveyed families was shared care by parents and grandparents (56.5%), followed by parental care (27.7%). Other care, such as by relatives or neighbors, was less common (5.1%). The surveyed parents were predominantly aged between 18–29 years (43.6%) and 30–39 years (49.3%). Over half of these parents had tertiary education (65.8%), which entails having completed an undergraduate, graduate, or postsecondary degree, while a smaller proportion had secondary education (18.9%). Most were from dual-income families (63.2%), where both father and mother were employed, with a minority from families where only the father worked (30.8%). A majority expressed no desire for further childbirth (67%). Most of these parents had only one infant (94%), with the majority of children aged between 2–3 years (39.1%). The distribution of male (50.3%) and female (49.7%) infants was almost equal, as shown in Table 2.

**Table 2:** Characteristics of the study sample.

Variable	*N*	preference for childcare		X^2^ value	*p*-value
		No	Yes		
Total	3,921	1856	2065		
Characteristics of families					
Residence				315.04	<0.001
Urban	2,322	828 (44.6%)	1,494 (72.3%)		
Rural	1,599	1,028 (55.4%)	571 (27.7%)		
Income				99.80	<0.001
<4,000	1,422	803 (43.3%)	619 (30.0%)		
4,000–8,000	1731	786 (42.3%)	945 (45.8%)		
>8,000	768	267 (14.4%)	501 (24.2%)		
Care model				35.25	<0.001
Parental care	1,087	565 (30.4%)	522 (25.3%)		
Grandparent care	420	206 (11.1%)	214 (10.4%)		
Shared care	2,215	1,025 (55.3%)	1,190 (57.6%)		
Other care	199	60 (3.2%)	139 (6.7%)		
Characteristics of parents					
Parents’ age				42.21	<0.001
18–29	1709	897 (48.3%)	812 (39.3%)		
30–39	1935	863 (46.5%)	1,072 (51.9%)		
≥40	277	96 (5.2%)	181 (8.8%)		
Education				221.72	<0.001
Primary/vocational	600	420 (22.6%)	180 (8.7%)		
Secondary	742	423 (22.8%)	319 (15.4%)		
Tertiary	2,579	1,013 (54.6%)	1,566 (75.9%)		
Employment status				193.60	<0.001
Both	2,478	970 (52.3%)	1,508 (73.0%)		
Just father	1,206	760 (40.9%)	446 (21.6%)		
Just mother	111	52 (2.8%)	59 (2.9%)		
Neither	126	74 (4.0%)	52 (2.5%)		
Reproductive will				0.81	0.6676
Willing	361	179 (9.6%)	182 (8.8%)		
Unwilling	2,629	1,239 (66.8%)	1,390 (67.3%)		
Not sure	931	438 (23.6%)	493 (23.9%)		
Characteristics of infants					
Infants’ number				6.76	0.0340
One	3,684	1762 (94.9%)	1922 (93.1%)		
Two	221	86 (4.7%)	135 (6.5%)		
More than two	16	8 (0.4%)	8 (0.4%)		
Infants’ age				191.67	<0.001
0–1	1,059	624 (33.6%)	435 (21.1%)		
1–2	1,328	712 (38.4%)	616 (29.8%)		
2–3	1,534	520 (28.0%)	1,014 (49.1%)		
Infants’ gender				0.01	0.9207
Male	1949	921 (49.6%)	1,028 (49.8%)		
Female	1972	935 (50.4%)	1,037 (50.2%)		

Chi-square test assessed sociodemographic factors stratified by preference for childcare. Factors such as residence, income, care model, parents’ age, education, employment status, and the number and age of infants were found to be statistically significant (*p* < 0.05).

### 3.2 Related sociodemographic factors of preference for childcare

Based on the results of the Chi-square test, statistically significant factors (*p* < 0.05) such as residence, income, care model, parents’ age, education, employment status, and the number and age of infants were included in the logistic regression model for binary logistic regression analysis.

In terms of characteristics of families, parents in rural areas are less likely to opt for child-care services compared to those in urban areas (OR = 0.478, 95%CI: 0.408–0.560). Parents with a monthly income above 4,000 CNY are more likely to choose childcare services compared to those earning less, and the higher the income (OR = 1.342, 95%CI: 1.090–1.652), the stronger the preference for childcare. Compared to parental care, grandparental care, shared care, and other care models show significant differences, but with parents under other care models (OR = 1.802, 95%CI: 1.257–2.583) showing a higher preference for childcare.

Regarding characteristics of parents, older parents (OR = 1.488, 95%CI: 1.112–1.991) are more inclined to opt for childcare services than younger ones; parents with medium and higher education levels are significantly more inclined toward childcare compared to those with lower education, with the preference increasing with higher educational attainment (OR = 1.966, 95%CI: 1.549–2.495). Dual-income families show a stronger preference for childcare compared to single-income (OR = 0.603, 95%CI: 0.499–0.729; OR = 0.799, 95%CI: 0.529–1.205) or non-working families (OR = 0.646, 95%CI: 0.433–0.965).

In terms of the characteristics of infants, parents with two or more children (OR = 1.392, 95%CI: 1.027–1.886; OR = 1.367, 95%CI: 0.474–3.945) are more inclined toward childcare than those with only one child. Furthermore, parents of children aged 1–2 and 2–3 years are more willing to choose childcare services compared to those with children aged 0–1 year, with the desire for childcare increasing as the child approaches the age of three (OR = 2.580, 95%CI: 2.162–3.077), as shown in Table 3.

**Table 3:** Logistic regression analysis results.

Factor	S.E	*Z*	*p*-value	OR, 95%CI
Characteristics of families				
Residence (Ref = urban)				
Rural	0.039	−9.14	<0.001***	0.478 (0.408–0.560)
Income (Ref < 4,000)				
4,000–8,000	0.090	1.27	0.205	1.108 (0.945–1.299)
>8,000	0.142	2.77	0.006**	1.342 (1.090–1.652)
Care model (Ref = Parental care)				
Grandparent care	0.095	−2.54	0.011*	0.714 (0.550–0.926)
Shared care	0.073	−2.45	0.014*	0.801 (0.671–0.956)
Other care	0.331	3.20	0.001**	1.802 (1.257–2.583)
Characteristics of parents				
Parents’ age (Ref = 18–29)				
30–39	0.076	0.29	0.768	1.022 (0.884–1.182)
≥40	0.221	2.67	0.007**	1.488 (1.112–1.991)
Education (Ref = Primary/vocational)				
Secondary	0.185	3.11	0.002**	1.476 (1.154–1.887)
Tertiary	0.239	5.55	<0.001***	1.966 (1.549–2.495)
Employment status (Ref = both)				
Just father	0.058	−5.23	<0.001***	0.603 (0.499–0.729)
Just mother	0.167	−1.07	0.285	0.799 (0.529–1.205)
Neither	0.132	−2.13	0.033*	0.646 (0.433–0.965)
Characteristics of infants				
Infant number (Ref = one)				
Two	0.216	2.13	0.033*	1.392 (1.027–1.886)
More than two	0.739	0.58	0.563	1.367 (0.474–3.945)
Infant age (Ref = 0–1)				
1–2	0.109	2.34	0.019*	1.231 (1.034–1.465)
2–3	0.232	10.54	<0.001***	2.580 (2.162–3.077)

***p < 0.001, **p < 0.01, *p < 0.05.

### 3.3 Childcare preferences and SDCS

Table 4 presents the results of descriptive analyses and t-tests related to SDCS. It indicates that parents of infants with different childcare preferences have varying needs for childcare services, with significant differences observed across all 9 dimensions, including environmental facilities, reception services, nursing care, early education, safety management, teacher professionalism, convenience services, home instruction content, and home instruction approach (*p* < 0.001). Furthermore, in each dimension, parents with childcare preferences scored higher than those without, this indicates that parents with a preference for childcare services have a higher demand for such services.

**Table 4:** Association of preference for childcare with SDCS.

Variables	Mean		*t*	*p*-value
	No	Yes		
Environmental facilities	21.90	23.20	9.66	<0.001
Reception services	24.30	26.82	14.34	<0.001
Nursing care	25.84	27.76	11.67	<0.001
Early education	17.16	18.49	12.31	<0.001
Safety management	22.14	23.42	10.01	<0.001
Teacher professionalism	31.36	33.02	9.63	<0.001
Convenience services	16.57	17.77	10.88	<0.001
Home instruction content	25.98	27.48	9.52	<0.001
Home instruction approach	23.63	25.04	7.96	<0.001

SDCS, the scale of demand for childcare services.

### 3.4 Results of binomial logistic regression analysis

Table 5 further analyzes factors in the SDCS that influence the childcare preference for childcare of parents. Three factors—reception services (OR = 1.10, *p* < 0.001), early education (OR = 1.10, *p* < 0.001), and convenience services (OR = 1.06, *p* < 0.001)—have a positive influence on parents’ preference for childcare. Conversely, environmental facilities have a negative impact on parents’ preference for childcare (*p* = 0.026, OR = 0.97), meaning that the higher the quality environment of a childcare institution, the less inclined parents are to utilize its services for their children. However, nursing care, safety management, teacher professionalism, home instruction, and home instruction approach have no significant influence on parents’ preference for childcare.

**Table 5:** Binomial logistic regression analysis results of SDCS.

Variables	OR	95%CI	P-Value
Environmental facilities	0.97	0.94–1.00	0.026*
Reception services	1.10	1.07–1.12	<0.001***
Nursing care	1.00	0.97–1.04	0.805
Early education	1.10	1.05–1.15	<0.001***
Safety management	0.96	0.91–1.01	0.140
Teacher professionalism	0.99	0.95–1.02	0.431
Convenience services	1.06	1.03–1.10	<0.001***
Home instruction content	0.98	0.95–1.01	0.155
Home instruction approach	1.00	0.98–1.02	0.767

***p < 0.001, **p < 0.01, *p < 0.05. SDCS, the scale of demand for childcare services.

### 3.5 Construction of decision tree model

**Figure 1:**
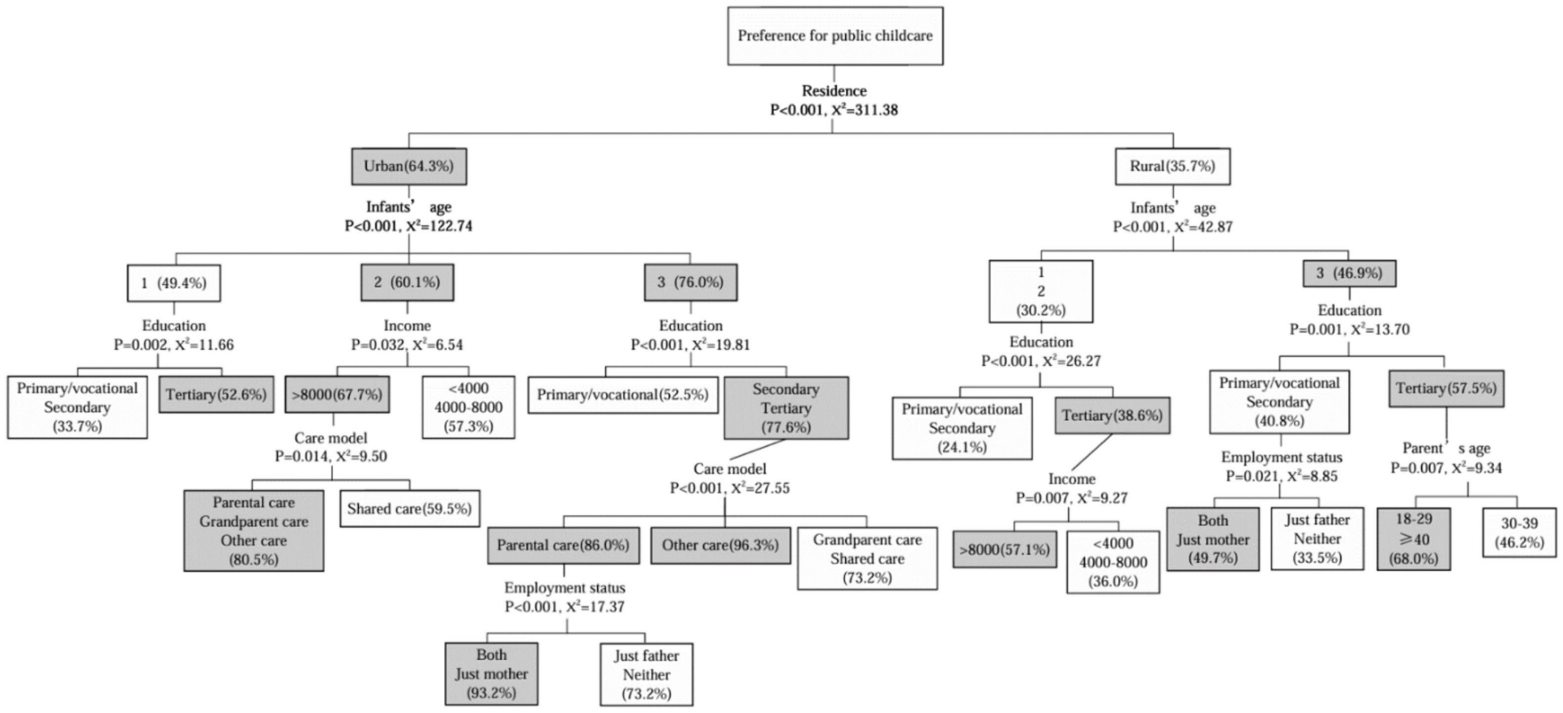
Classification and regression tree model.

The results of the decision tree model are shown in Figure 1. The decision tree model has 5 layers, including 30 leaf nodes, with a misclassification rate of 26.7%. The preference for childcare is mainly related to factors such as residence, infant’s age, income, education, care model, employment status, and parents’ age. The decision tree model shows that the root node variable is ‘residence’, indicating that residence is the most important factor affecting parents’ childcare preference. The decision tree model primarily has two branch paths: urban and rural. Among them, the preference for childcare among urban parents is 64.3%, and for rural parents, it is 35.7%, with the rate in urban areas being 1.8 times that of rural areas, as detailed in Figure 1.

A closer examination of both the urban and rural branches of the tree highlights the age of the infant as a critical determinant in childcare preference. For parents with children aged 2–3 years in urban settings, childcare preference peaks at 76%, outstripping other groups at 60.1 and 49.4%. Similarly, in rural areas, parents with children in the same age group show a higher inclination (46.9%) compared to their counterparts (30.2%). Additionally, the level of education attained by parents in urban areas significantly impacts their childcare choices. Those with higher education levels exhibit a greater propensity (77.6%) for childcare compared to those with lower educational qualifications (52.5%). In situations where children are under alternative care arrangements, the preference for childcare escalates to 96.3%, notably surpassing the preference of parents who rely on parental (86%) or grandparental and joint care (73.2%). For educated rural parents, those with higher education demonstrate a higher inclination (57.5%) toward childcare than those with middle or lower education levels (40.8%).

### 3.6 Evaluation of decision tree model

A 10-layer cross-validation model was used to identify model accuracy, which was 73.3%, indicating that the model was effective, as detailed in Table 6.

**Table 6:** The accuracy of the model.

Name	*n*	%
Model accuracy	2,874	73.3%
Model error	1,047	26.7%
Total	3,921	100

## 4 Discussion

In recent years, based on consideration of China’s population strategy and practical problems, the Chinese government has increasingly focused on the development of the infant and toddler childcare service industry. We conducted a cross-sectional survey on the preference for childcare among parents of 0-3-year-old children in Chongqing, China, aiming to analyze the factors influencing this preference and understand the childcare service needs of these parents. This study found that 52.7% of parents had a preference for childcare, while 47.3% do not. Factors influencing parents’ preferences include residence, infant’s age, income, education, childcare model, employment status, parent’s age, reception services, early education, convenience services, and environmental facilities in SDCS.

### 4.1 Characteristics of families and preferences for childcare

Our study found that residence influences parents’ decisions, the results of the decision tree show that residence is the most important influencing factor of preference for childcare. Those living in rural areas are less inclined to use childcare services compared to urban residents, consistent with previous research (Li et al., 2017). This could be due to several reasons. For one, resources in rural areas, including talent, financial support, and types of services for the childcare industry, are more scarce compared to urban areas (Hao et al., 2014). Additionally, urban women might face more pronounced conflicts between work and childcare, necessitating external support (Hung and Chung, 2001), this external support can be in the form of professional childcare services or grandparent care. Although grandparents can take care of their children, taking care of them is not always pleasant, and grandparents may feel physically exhausted, have less free time, etc., due to the work of taking care of them (Goodfellow, 2003; Goh, 2009; Lo and Lindsay, 2022). Besides, urban parents generally have stronger financial and payment means, if they do not want to put too much pressure on their grandparents, they can choose childcare (Yu and Zhao, 2021; Chen et al., 2023). Evidently, family income is a significant factor, with higher income correlating positively with the preference to choose childcare services. A higher income level implies stronger financial capacity to afford childcare.

The childcare model significantly impacts parents’ choice of childcare services. Compared to parental care, shared care and grandparental care models show a lower inclination toward childcare services, whereas other care models show a higher inclination. Shared care refers to the shared responsibility between parents and grandparents in postnatal childrearing (Xiao and Loke, 2022). In China, grandparental care, regardless of whether the grandparents live with the family, is a common and increasing phenomenon (Li et al., 2017). This care model, unaffected by childcare institutions or community childcare resources, indirectly proves to be a result of intergenerational solidarity. Parents tend to trust grandparental assistance over childcare services, hence showing lower preference toward childcare services in such cases (Chen et al., 2011). Other care models, like those provided by neighbors or nannies, are more likely to be replaced by childcare services.

### 4.2 Characteristics of parents and preference for childcare

The likelihood of choosing childcare services increases with the parents’ age, reducing some of their own pressure. Education level is one of the most important factors influencing childcare preference. Parents with higher education levels are willing to spend more time caring for infants and are more inclined to choose formal childcare services (Kalil et al., 2012; Carlin et al., 2019). This may be because more educated parents are more receptive to advanced, scientific parenting concepts, focusing more on fostering good habits in infants, providing scientific training and proper guidance, planning activities that suit children’s developmental needs, and reducing health risks for children (Mangrio et al., 2011; Kalil et al., 2012). Dual-income families have higher economic income compared to single-income families, offering more choices in various services. This explains why the preference for childcare is lower in families where only the father works, as full-time mothers provide care that is more reassuring than childcare institutions and has fewer childcare costs.

### 4.3 Characteristics of infants and preference for childcare

The number and age of infants also influence parents’ preferences for childcare services. Caring for more than one infant increases the complexity of care, requiring more time and energy from parents and dealing with additional challenges such as managing sibling relationships, which adds to the parental burden (Qian et al., 2021; Hong et al., 2023). As infants grow older, their parents are more willing to choose childcare services, consistent with previous research (Ferguson et al., 2020). This could be due to concerns about physical development, psychological health, and safety. Infants and toddlers who are too young are more vulnerable, and for safety reasons, most parents are reluctant to opt for childcare when infants and toddlers are too young (Yang, 2018). Studies have shown that if childcare is chosen early, it can increase the financial burden on parents (Chen et al., 2023). Additionally, research indicates that child-care institutions in China mostly accept children over two years old, with a lack of supply for younger infants, thus limiting parents’ options (Li et al., 2022).

### 4.4 SDCS and preference for childcare

In the childcare service demand scale section, we also derived some meaningful results. Parents of infants prefer childcare institutions that offer reception services, early education, and convenience services, mainly because these services meet their dual needs as both working professionals and caregivers. These choices are typically made to balance their children’s educational and developmental needs with the family’s daily routine. Parents consider their children’s best interests when choosing childcare services, including a high-quality educational and care environment, while also taking into account their own work and life requirements, such as the childcare center’s location and convenient operating hours. These decisions reflect the parents’ emphasis on early childhood education and their efforts to balance family and work responsibilities.

The environmental facilities can hinder parents’ choice of childcare services, primarily due to cost and affordability issues. In China’s current childcare market, private institutions are more numerous (Chen et al., 2023), indicating that better facilities come at a higher cost. However, childcare expenses significantly influence parents’ preference to opt for childcare services, with most parents not willing to invest excessively in this area. Thus, while high-quality early education and childcare services are seen as a good investment, their cost can become an unaffordable burden for families (Ferguson et al., 2020). Additionally, parents might not be able to fully express their preferences for childcare services, and the actual decisions made may differ from their stated preferences. This means that even though parents may ideally wish to choose better-quality childcare services, their actual choices may be constrained by cost and other practical limitations.

As a result, early education programs should be included in affordable, convenient childcare services that are demand-driven and developed in different regions. For instance, childcare facilities can collaborate with community service centers across different areas to offer childcare services at reduced costs and to benefit the local community. As they grow, childcare facilities should focus on safety concerns, strengthen their emergency response strategy for all types of emergencies, prevent the occurrence of harmful accidents, and gain the trust of new parents. Create a coordinated development model centered on early education, use the childcare service concept of “integration of protection and education,” and innovate the early education service model. Parents of infants and toddlers could also improve childcare awareness, such as through seminars or websites.

## 5 Conclusion

This study found that half of the parents had a preference to use childcare services, parents with higher incomes, higher education levels, older ages, multiple infants, and dual-career living in urban areas have a stronger preference for childcare. In the shared or grandparent care model, the childcare preference is not obvious. Parents of infants tend to choose childcare institutions that provide reception services, early education, and convenience services. Higher-quality environmental facilities tend to reduce the preference of parents for childcare. Therefore, more convenient and low-cost childcare services should be developed, and more childcare options should be provided to reduce the cost of caring for parents. Some benefits should be provided to families with infants to ease the financial burden on parents. In addition, relevant childcare knowledge should be vigorously disseminated.

Our study has certain limitations. This survey was conducted in Chongqing, China, and may not fully represent the situation nationwide. Additionally, being a cross-sectional survey, it does not reflect the changes in the needs of parents of infants for childcare services over time. Participation is also limited to the use of online services, which may not include a subset of groups. The study was conducted during the COVID-19 pandemic, and respondents’ preferences may have been affected by COVID-19, but we did not reflect this factor in the questionnaire.

## Data Availability

The raw data supporting the conclusions of this article will be made available by the authors, without undue reservation.

## Footnotes

^1^https://www.wjx.cn/
